# The Ruby UCSC API: accessing the UCSC genome database using Ruby

**DOI:** 10.1186/1471-2105-13-240

**Published:** 2012-09-21

**Authors:** Hiroyuki Mishima, Jan Aerts, Toshiaki Katayama, Raoul J P Bonnal, Koh-ichiro Yoshiura

**Affiliations:** 1 Department of Human Genetics, Nagasaki University Graduate School of Biomedical Sciences, 1-12-4 Sakamoto, Nagasaki, Nagasaki, 852-8523, Japan; 2 Faculty of Engineering – ESAT/SCD, Leuven University, Kasteelpark Arenberg 10 – bus 2446, 3001, Leuven, Belgium; 3 IBBT-KULeuven Future Health Department, Leuven, Belgium; 4 Database Center for Life Science, Research Organization of Information and Systems, Faculty of Engineering Bldg. 12, The University of Tokyo, 2-11-16, Yayoi, Bunkyo-ku, Tokyo, 113-0032, Japan; 5 Integrative Biology Program, Fondazione Istituto Nazionale di Genetica Molecolare, via Francesco Sforza, 28-20122, Milan, Italy

## Abstract

**Background:**

The University of California, Santa Cruz (UCSC) genome database is among the most used sources of genomic annotation in human and other organisms. The database offers an excellent web-based graphical user interface (the UCSC genome browser) and several means for programmatic queries. A simple application programming interface (API) in a scripting language aimed at the biologist was however not yet available. Here, we present the Ruby UCSC API, a library to access the UCSC genome database using Ruby.

**Results:**

The API is designed as a BioRuby plug-in and built on the ActiveRecord 3 framework for the object-relational mapping, making writing SQL statements unnecessary. The current version of the API supports databases of all organisms in the UCSC genome database including human, mammals, vertebrates, deuterostomes, insects, nematodes, and yeast.

The API uses the bin index—if available—when querying for genomic intervals. The API also supports genomic sequence queries using locally downloaded *.2bit files that are not stored in the official MySQL database. The API is implemented in pure Ruby and is therefore available in different environments and with different Ruby interpreters (including JRuby).

**Conclusions:**

Assisted by the straightforward object-oriented design of Ruby and ActiveRecord, the Ruby UCSC API will facilitate biologists to query the UCSC genome database programmatically. The API is available through the RubyGem system. Source code and documentation are available at https://github.com/misshie/bioruby-ucsc-api/ under the Ruby license. Feedback and help is provided via the website at http://rubyucscapi.userecho.com/.

## Background

The University of California, Santa Cruz (UCSC) genome database [1] is one of the most common gateways to access genomic sequence and annotation data of humans and other organisms. Besides a web-based genome browser [2], the database is programmatically accessible through three interfaces: the official command-line tools and libraries [3,4], the Distributed Annotation System (DAS) [5] server, and direct access to a public MySQL database server. UCSC’s official tools consist of command-line executables and API libraries written in the C language. The C API widely supports the functionality of the database with good performance. These tools and libraries are available at the Kent source tree [3,6]. The UCSC DAS server, which supports previous DAS version 0.95, offers a simple interface for programmatic access to the database. However, it has a limitation in supported types of annotations and has disadvantages in its performance. The public MySQL server, finally, offers access to almost the same up-to-date database for the genome browser but requires the user to program raw SQL statements. Given the pervasive use of scripting languages in this field of research, there is a significant demand for simple APIs that allow construction of automated queries in these languages. In particular, the Ruby programming language has been widely adopted in the bioinformatics domain [7,8]. Libraries including BioRuby [9] and the Ruby Ensembl API [10] have shown the value of database APIs for Ruby.

Here, we describe the Ruby UCSC API, an API to query the UCSC genome database.

## Implementation

### Object-relational mapping

**Figure 1:**
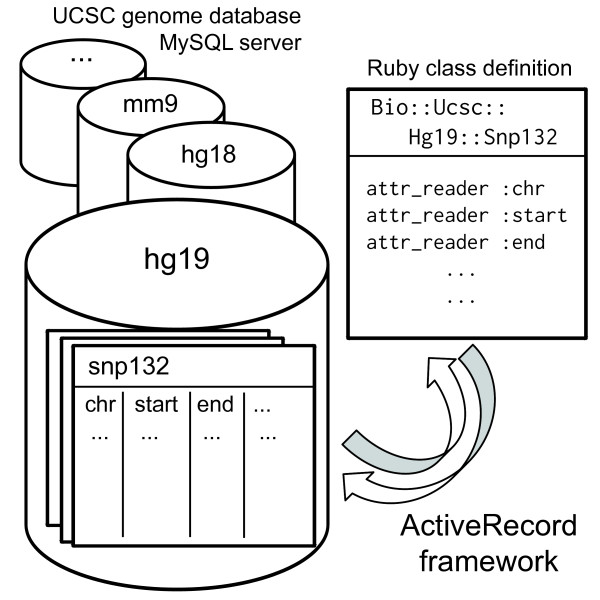
**Overview of the Ruby UCSC API.** The UCSC genome database provides a public MySQL server consisting of multiple databases, e.g. mm9, hg18, and hg19. Each database contains many tables. The Ruby UCSC API and the ActiveRecord framework automatically define classes and instance methods corresponding to tables and their fields, respectively.

The Ruby UCSC API is based on the ActiveRecord 3 framework—a component of Ruby on Rails [11] —for the object-relational mapping (Figure 1). A database in the UCSC genome database is represented as a module under the Bio::Ucsc name space and a table in the database is represented as a class (subclass of the ActiveRecord::Base class) under the database module. For example, the “snp132” table in the human genome assembly “hg19” database is referred to as Bio::Ucsc::Hg19::Snp132. Query APIs to a table are automatically defined from the database schema as class methods following the ActiveRecord’s method naming convention. For example, if the “snp132” table has a field (column) “name”, the Snp132.find_by_name method is readily available. Records (rows) are instances of the corresponding table class, for which values of any field can be obtained.

### Dynamic class definition

The UCSC database is optimized to serve the genome browser, resulting in a very large number of tables (about 41,840 tables as MySQL *.MYD files) for which the API has to provide access. Furthermore, these database components are updated frequently. Static definitions of many table classes would make API code maintenance difficult. Therefore, we employed dynamic class definition in the Ruby UCSC API. When a table is referred to for the first time, the API fetches the database schema of that table to determine the data types and then creates an appropriate Ruby class for that table. This lazy generation of the classes also contributes to accelerate the initialization of this API when compared to having static classes for thousands of tables.

### Supporting auxiliary flat files

A subset of the UCSC genome database, including genome sequences, is not stored in the MySQL database but needs to be downloaded locally for access. The Ruby UCSC API offers methods to access these downloaded genome sequences (*.2bit files).

### Dependencies and environment

The Ruby UCSC API depends on ActiveRecord 3 and is designed as a BioRuby plugin using the Biogem system [12,13], which organizes RubyGems packages and their dependencies for the BioRuby library.

The Ruby UCSC API is written purely in Ruby. This increases the compatibility of the API for various operating systems and implementations of the Ruby interpreter. The API currently supports different Ruby interpreters including Ruby version 1.9.2 or later, Ruby version 1.8.7 or later, and JRuby 1.6.3 or later.

## Results and discussion

### Features and usage

**Figure 2:**
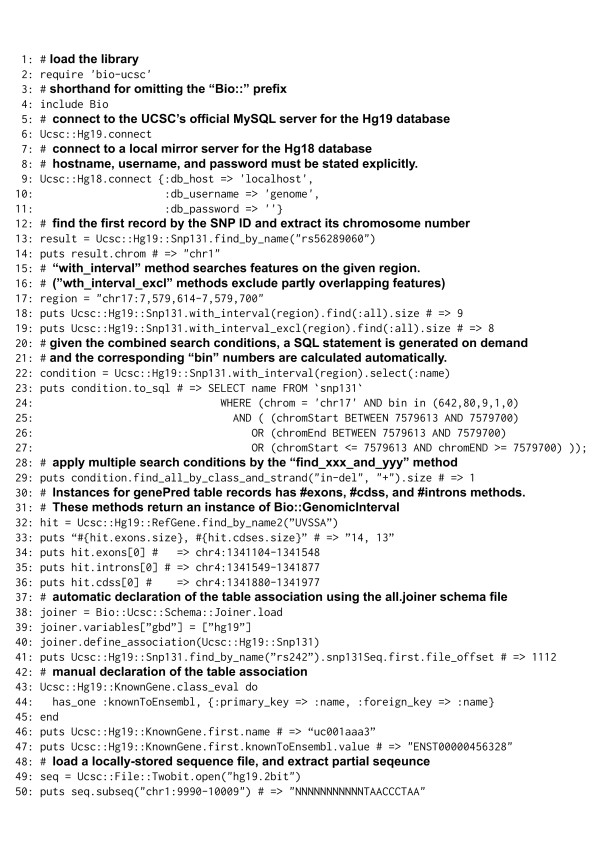
Example script using Ruby UCSC API.

Figure 2 shows examples of the Ruby UCSC API in use.

#### Database connection

After loading the Ruby UCSC API library (line 2), a connection to a database can be established by the ‘connect’ method (line 6). While the default connection is made against the UCSC public MySQL server, alternative full or partial mirror servers can be used as well (line 9–11). The API can connect to multiple databases simultaneously.

#### Table query

Users can query the database by series of “find” class methods which are dynamically defined for each table class by the ActiveRecord. First of all, find_by_[field-name] and find_all_by_[field-name] class methods retrieve the first or all matching records, respectively. Example queries for the “name” field are shown in line 13. Multiple conditions joined by the _and_ operator are also accepted (line 29). According to the ActiveRecord’s convention, values of the other fields in a retrieved record can be referred to by using instance methods denoted by the field names (line 14).

#### Query by genomic intervals

A genomic interval can be expressed by a string like “chr1:123,456-456,789” as used in the graphical web interface of the UCSC Genome Browser (line 17). An interval query condition is passed by the with_interval method (line 18–19). This method automatically absorbs the difference of genomic coordinate conventions between intuitive 1-based coordinates and database internal 0-based coding system (compare line 17 and 25–27). The with_interval method allows retrieving all features that are overlapping with the given interval (line 18). Instead, the with_interval_excl method only returns features that lie completely within the region and features partially overlapping with the region are excluded (line 19).

#### Bin indexing system

To achieve high query performance for large tables, the UCSC database uses a bin indexing system [6]. In this system, genomic positions in a chromosome are separated into hierarchies of bins that are sized into 512Mbase, 64Mbase, 8Mbase, 1Mbase and 128kbase. Any annotation in a genomic interval is stored in the minimum sized bin that encompasses the whole interval. For a genomic interval query, if the target table has a “bin” field, the API automatically calculates a list of bins that potentially contain annotations for the interval and applies the list to generate an SQL statement to narrow the target record. This is a key feature of the API because multiple queries for genomic intervals without using the bin index take excessive times, especially for large tables such as dbSNP.

#### Building SQL statements

Methods to specify search conditions, such as with_interval, select, where, order, limit and group, can be combined by chaining (line 22–27). When a find method or one of the methods to access arrays (such as find_all, first and []) is called for the condition, the constructed SQL query is executed and the results are returned (line 29).

#### Methods to access information of exons, CDSs, and introns

Instances of “genePred” table classes, such as RefGene, EnsGene, and KnownGene, have exons, cdss, and introns methods. These methods return arrays of Bio::GenomicInterval objects sorted according to the gene strand (line 32–36).

#### Table association

The joiner schema file describes the links between the tables of the UCSC genome database. The Bio::Ucsc::Schema::Joiner.load class method takes an URI of the schema file. If the URI is not given, UCSC’s all.joiner file [14] is used (line 38). The format of the joiner file is documented in the Kent source tree [15]. Variables in the joiner file can be overwritten. For example, overwriting the gbd variable that stores whole databases can restrict databases used for the link search (line 39). The define_association method takes a table class and defines all the associations of given table (line 40). Unconnected databases and undefined tables are ignored during definition. Linked results are always returned as an array (line 41). The table association also can be defined manually. When a record in a table can be joined with a record of another table by sharing the same value (foreign key), the has_one / has_many methods are used to declare the association (line 43–45). Once the table association is declared, a table can refer to the associated table using a method of its record object (line 46–47).

#### Retrieval of genomic sequences

Extraction of genomic sequences in the given genomic intervals is a frequent task. The UCSC genome database does not store the genomic sequences in the MySQL databases. Instead, they provide the sequences as *.2bit files. These files are usually processed by UCSC’s tools written in C. To improve the compatibility, we implemented the same functionalities in Ruby. With the Bio::Ucsc::File::Twobit class, *.2bit files are interpreted in Ruby and subsequences can be extracted by the subseq method (line 49–50).

### Current limitations

The current version of Ruby UCSC API uses information of the joiner schema file to find table associations. The all.joiner file, however, describes additional information of including which tables are chromosome- rather than genome-based, field values that have to be transformed to define table associations, and tables with exceptional structures. In future versions, the API will use this information to make user scripts simpler and to follow database structure updates immediately. So far, manual definition of table associations still has an advantage in performance by minimizing table association definitions, especially in some tables that have complicated associations.

For some tables including subsets of the Encyclopedia of DNA Elements (ENCODE) [16], the actual data are not stored in the MySQL database itself but are stored as references to BigWig, BigBed [17] and BAM [18] files. BigWig and BigBed can be accessed by the UCSC tools in C. BAM files can be processed by third-party tools such as Samtools [18], and Picard [19]. To date, the Ruby UCSC API does not support these yet, however, users can use the bio-samtools BioRuby plugin [20] for these tasks.

### Existing UCSC APIs for scripting languages

APIs for the UCSC genome database using scripting languages are still limited. For Perl, the Genoman module [21] offers interfaces to databases including the UCSC genome database. For Python, the Cruzdb library [22] offers an SQLAlchemy-based API for the UCSC genome database. The biggest advantage of Ruby UCSC API described here is that Ruby and the Active Record framework enable simplified query and retrieved record description. Moreover, the Ruby UCSC API does not depend on UCSC’s command-line tools. This makes its installation easier and increases interoperability for various environments including a Java-based Ruby interpreter, JRuby.

## Conclusions

UCSC’s official executables and C libraries are the most comprehensive and fastest API for the UCSC genome database; however, APIs for scripting languages still have significant advantages for users because their concern is not only a runtime speed but also a total time required for the programming to obtain the results. The Ruby UCSC API offers effective productivity and can therefore have a significant impact in the field.

The Ruby UCSC API already supports all organisms in the UCSC genome database (Table 1). In future releases, more comprehensive supports for new organisms and older or updated genome assemblies will be added.

**Table 1:** Supported databases

**Clade/organism**	**Databases**
human	Hg19, Hg18
mammals	chimp (PanTro3), orangutan (PonAbe2), rhesus (RheMac2), marmoset (CalJac3), mouse (Mm9), rat (Rn4), guinea pig (CavPor3), rabbit (OryCun2), cat (FelCat4), panda (AilMel1), dog (CanFam2), horse (EquCab2), pig (SusScr2), sheep (OviAri1), cow (BosTau4), elephant (LoxAfr3), opossum (MonDom5), platypus (OrnAna1)
vertebrates	chicken (GalGal3), zebra finch (TaeGut1), lizard (AnoCar2), X. tropicalis (XenTro2), zebrafish (DanRer7), tetraodon (TetNig2), fugu (Fr2), stickleback (GasAcu1), medaka (OryLat2), lamprey (PetMar1)
deuterostomes	lancelet (BraFlo1), sea squirt (Ci2), sea urchin (StrPur2)
insects	D.melanogaster (Dm3), D.simulans (DroSim1), D.sechellia (DroSec1), D.yakuba (DroYak2), D.erecta (DroEre1), D.ananassae (DroAna2), D.pseudoobscura (Dp3), D.persimilis (DroPer1), D.virilis (DroVir2), D.mojavensis (DroMoj2), D.grimshawi (DroGri1), Anopheles mosquito (AnoGam1), honey bee (ApiMel2)
nematodes	C.elegans (Ce6), C.brenneri (CaePb3), C.briggsae (Cb3), C.remanei (CaeRem3), C.japonica (CaeJap1), P.pacificus (PriPac1)
others	sea hare (AplCal1), yeast (SacCer2)
common databases	Go, HgFixed, Proteome, UniProt, VisiGene

The Ruby UCSC API is freely available as a Rubygem package. Source code and documentations are also available at https://github.com/misshie/bioruby-ucsc-api/. Documentation and feedback are available at the UserEcho site at http://rubyucscapi.userecho.com/.

### Availability and requirements

**Project name**: The Ruby UCSC API

**Project home page**: https://github.com/misshie/bioruby-ucsc-api

**Feedback and help**: http://rubyucscapi.userecho.com/

**Operation systems**: Platform independent

**Programming language**: Ruby

**Other requirements**: Ruby interpreter (Ruby 1.8.7 or later, Ruby 1.9.2 or later, or JRuby 1.6.3 or later), and ActiveRecord (version 3.0.7 or later).

**License**: The Ruby License

**Any restrictions to use by non-academics:** none

## Competing interests

The authors declare that they have no competing interests.

## Authors' contributions

HM and JA conceived the project and implemented the API. TK, RJPB and KY reviewed the API design and implementation. HM, JA, TK, RJPB and KY wrote the manuscript. All authors read and approved the final manuscript.
